# Obesity and Dental Caries in School Children

**DOI:** 10.3390/jcm13030860

**Published:** 2024-02-01

**Authors:** Amir Mohajeri, Gabrielle Berg, April Watts, Val Joseph Cheever, Man Hung

**Affiliations:** 1 College of Dental Medicine, Roseman University of Health Sciences, South Jordan, UT 84095, USA; gberg274@student.roseman.edu (G.B.); awatts447@student.roseman.edu (A.W.); jcheever@roseman.edu (V.J.C.); mhung@roseman.edu (M.H.); 2 George E. Wahlen Department of Veterans Affairs Medical Center, Salt Lake City, UT 84148, USA; 3 Huntsman Cancer Institute, Salt Lake City, UT 84112, USA; 4 School of Medicine, University of Utah, Salt Lake City, UT 84113, USA

**Keywords:** obesity, dental caries, school children, oral health status, untreated dental caries, dental caries experience, oral health, dental health

## Abstract

(1) Background: Childhood obesity and dental caries are common chronic conditions with multiple contributing factors, linked to negative health consequences and significant expenses in healthcare. The aim of this study was to assess the correlation between obesity and dental caries in school-aged children; (2) Methods: Data from 3323 6–12-year-old children from the National Health and Nutrition Examination Survey (NHANES) 2011–2016 were analyzed. The NHANES was conducted at the Centers for Disease Control and Prevention (CDC) in the United States. The CDC standard was used to define obesity. Dental caries was measured during clinical examinations and summarized using DMFT scores for caries experience and prevalence (dt > 0) for untreated caries. The study examined the correlation between obesity and dental caries using regression models that considered demographic variables, family socioeconomic status, and the child’s intake of added sugars as controlling factors; (3) Results: The association between obesity and dental caries was not significant in either unadjusted or adjusted models; and (4) Conclusion: The data indicate that untreated caries and caries experiences are not directly correlated with childhood obesity. There are, however, common causes of poor dental health and childhood obesity: culture, poverty level, lifestyle, and family traditions and habits. Dentists must be aware of factors influencing the development of childhood caries so that they can intervene as early as possible.

## 1. Introduction

Obesity and dental caries are enduring and complex health conditions that impact an individual’s wellbeing over the course of their life, imposing significant financial burdens on the national healthcare system [1,2]. In 2016, over 340 million children and adolescents aged 5–19 years old were overweight or obese worldwide [3]. Obesity also affected about one in five school-aged children in the United States (US) in 2017–2018 [4]. In addition, the Centers for Disease Control and Prevention (CDC) showed that the prevalence of total and untreated dental caries in US school-aged children was 50.5% in 2015–2016 [5]. It reported dental caries prevalence in children and adolescents from 2011 to 2012, in which they provide the following findings: the rate of dental caries among those aged 6–8 was nearly 14%, while it was twice as high for those aged 9–11 in the United States [6]. 

There is still debate about the relationship between obesity and dental caries. Research in this area has grown rapidly in the past five years, with several systematic reviews published [7,8,9]. There is some evidence that obesity is positively associated with dental caries [9,10,11,12], but there is also some evidence that it is not [7,8,13]. The mixed findings highlight the need for further research.

Obesity and caries are of public health concern since they affect many children and adolescents worldwide. Both health concerns can have adverse impacts on school children’s wellbeing and quality of life, with an added side effect of societal costs [8]. Health problems associated with growth and development, and with oral disease, stem from complicated foundations; thus, there is a need to further explore how low levels of parental income and education may underlie this faulty foundation for both obesity and dental caries in young children [9]. Moreover, the impact that these social determinants has on a child’s dietary habits can directly relate to their oral health status [8].

Eating behavior and oral health share a synergistic yet intricate relationship. Diet, nutritional composition, and poor oral and dental conditions interact and have a role in caries formation. For example, sweet foods incorporated into an individual’s snacking patterns are the main factors of a cariogenic diet. Dietary imbalances can lead to varying degrees of malnutrition, resulting in the hypofunction of salivary glands as well as altered salivary composition and reduced buffering capacity. In addition, underlying poverty, inadequate birth spacing, unclean water, and low-quality food are factors that negatively influence children’s exposure to a balanced diet. The regional differences in the oral health disparities of children are directly affected by the quality, access, and cost of care, as well as the parent’s oral health education [14]. 

Some studies have suggested that obesity can modify oral microflora and salivary properties, which in turn may contribute to dental caries in children [15,16,17]. Based on this finding, dental caries could be a consequence of obesity. Most experts, however, believe that obesity and dental caries are related by common risk factors, such as low socioeconomic status and a high sugar intake [14,18]. Hence, the purpose of this study was to determine whether or not obesity and dental caries are related after controlling for demographic factors, family socioeconomic status, and the intake of added sugars.

## 2. Materials and Methods

### 2.1. Data Source

The current study used cross-sectional data from three cycles of the National Health and Nutrition Examination Survey (NHANES), 2011–2012, 2013–2014, and 2015–2016, aiming to increase the reliability and stability of estimates across subgroups. The NHANES collects data from a nationally representative sample of the non-institutionalized population in the US through a complex, multistage probability sampling design. Conducted biennially, each NHANES cycle includes interviews, physical examinations, and laboratory tests. Within the NHANES examination, various health-related measurements, such as body mass index (BMI) determination, are taken. Simultaneously, an oral health examination, encompassing assessments like dental caries examinations, occurs during the same cycle. This ensures that the determination of BMI and the analysis of oral health coincide in the NHANES examination. Both measurements are integral components of the comprehensive health assessment conducted during the survey, enabling researchers to investigate connections between oral health, body weight, and other health indicators in the U.S. population. According to the Ethics Review Board of the National Center for Health Statistics Research, the NHANES obtained ethical approval, with the following ethics approval numbers: Protocol #2011–17 (NHANES 2011–2012) and Continuation of Protocol #2011–17 (NHANES 2013–2016) [19]. Further, written parental permission was obtained for all participants who were minors. Approximately 10,000 individuals undergo home interviews and are evaluated for various health factors at a mobile examination center (MEC) in each cycle of the NHANES. Overall, there were 9756 participants in 2011–2012 (response rate: 72.6%), 10,175 in 2013–2014 (71.0%), and 9971 in 2015–2016 (61.3%) [20]. Detailed information about the NHANES is available on the CDC website [21].

**Figure 1:**
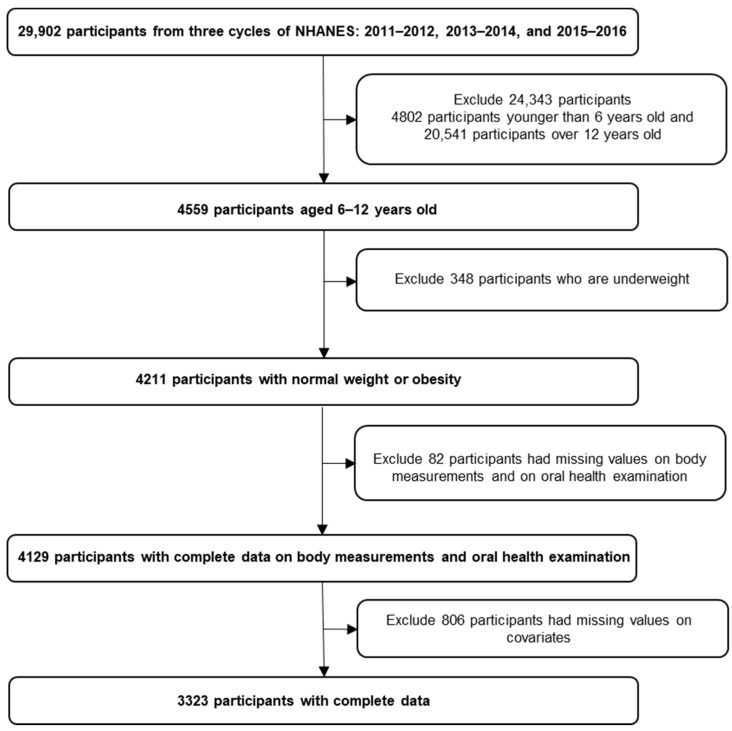
Selection criteria flowchart.

Inclusion/exclusion criteria: The study population was limited to children aged 6–12 years old because the study period encompasses important developmental, behavioral, and social factors that may influence oral health outcomes and is an ideal period for investigating the relationship between obesity and dental caries. In this analysis, participants who were underweight were excluded because increased body weight can be isolated and analyzed more accurately without the confounding effect of underweight conditions. Excluding underweight participants helps create a more homogeneous study population. This homogeneity allows for clearer comparisons and more accurate assessments of the relationship between obesity and dental caries by reducing variability associated with different weight categories. The analysis also excluded individuals with missing values for body measurements, oral health examinations, and covariates. Figure 1 displays the selection criteria flowchart.

### 2.2. Measures

#### 2.2.1. Outcome Measure

In the study, dental caries was determined by licensed dentists who had been trained in NHANES methods. An artificial light, a portable dental chair, and compressed air were used for the examinations at the MEC. Two dental caries measures were used in this study as outcomes: dental caries experience, measured using the decayed, missing and filled teeth (DMFT) index, and untreated dental caries, defined by the presence of at least one surface with a surface condition of 0–4 or at least one untreated carious root tip. The DMFT index was considered for permanent teeth, which are relevant for individuals beyond the age of 6 when primary dentition transitions to permanent dentition [22]. Permanent teeth are more accessible and may provide a more comprehensive understanding of long-term oral health. According to the ‘Coronal Caries: Tooth Count’ segment of the dental examination, the DMFT index was calculated as the total count of codes E, J, K, M, P, Q, R, T, X, and Z. Code E denotes missing due to dental disease, J indicates the presence of a permanent root tip without restoration or an attached device, K signifies a primary tooth with a carious surface condition, M represents missing due to other causes, P denotes missing due to dental disease but replaced by a removable restoration, Q indicates missing due to other causes but replaced by a removable restoration, R signifies missing due to dental disease but replaced by a fixed restoration, T denotes the presence of a permanent root tip with a restoration or attached device, X indicates missing due to other causes but replaced by a fixed restoration, and Z represents a permanent tooth with a carious surface. Caries scoring criteria used in the dental examination, along with quality assurance and training/calibration details, are described in-depth in the NHANES plan and operations manual [23].

#### 2.2.2. Independent Variable

The child’s body measurements were collected at the MEC by trained health technicians. Height was determined using a stadiometer equipped with an adjustable headpiece and a stable vertical backboard, while participants, attired in the standard MEC examination gown, were weighed on a digital scale [24]. The CDC standard was used to classify children based on height and weight. The CDC’s sex-specific 2000 BMI-for-age growth charts for the U.S. child population established normal weight as a BMI-for-sex-and-age equal to or greater than the 5th percentile but less than the 85th percentile. Overweight was defined as a BMI-for-sex-and-age equal to or higher than the 85th percentile but less than the 95th percentile, while obesity was indicated by a BMI-for-sex-and-age equal to or higher than the 95th percentile [25]. The obese and overweight groups were merged into one group for analysis. 

#### 2.2.3. Covariates

In the analysis, potential confounders of the association between obesity and dental caries included the child’s demographics, added sugar intake, and family socioeconomic status. Using the poverty income ratio, the socioeconomic status of families was estimated by dividing their income by the poverty guidelines specific to their household size, state, and year. Child demographic factors included age, sex, and race or ethnicity (Mexican American, other Hispanic, non-Hispanic white, non-Hispanic black, non-Hispanic Asian, and other race including multi-racial). In the MEC, a 24 h dietary recall interview was conducted to estimate the child’s dietary intake of all foods and beverages [26]. The Food Patterns Equivalent Database (FPED) of the US Department of Agriculture (USDA) was used to estimate added sugar consumption for each NHANES cycle [27]. This database expresses the content of added sugars in teaspoon equivalents (tsp. eq.). One teaspoon equivalent of added sugars is defined as 4.2 g of sugar, the amount of total sugars present in one teaspoon of granulated sugar. As defined by the FPED, added sugars are sugars, syrups, or caloric sweeteners added to foods and beverages during processing or home preparation, as well as sugars added at the table (e.g., adding sugar to coffee or tea) [28]. A child’s intake of added sugars was categorized into quartiles for analysis. 

#### 2.2.4. Statistical Analyses

Data analysis was conducted using SPSS version 29. The prevalence of untreated dental caries was analyzed with Chi-square tests, and sociodemographic factors were taken into account. Additionally, DMFT scores were subjected to comparison with sociodemographics using the Mann–Whitney U test or Kruskal–Wallis test (as appropriate). Obesity prevalence was also assessed in relation to sociodemographics using the Chi-square test. A one-way ANOVA test was used to determine if there was a difference in mean age between boys and girls.

Logistic regression models were used to examine the relationship between obesity and untreated caries. Exponentiated coefficients from these models were presented as odds ratios (OR) with their respective 95% confidence intervals. Generalized linear regression models were used to evaluate associations of obesity and caries experience, modeled as a scale variable. The coefficients of these models were also exponentiated and presented as rate ratios (RR) with respective 95% confidence intervals.

The initial regression analysis explored the link between obesity and untreated dental caries (Model 1). In Model 2, socio-demographic factors such as sex, age, and race/ethnicity were adjusted for. In Model 3, family socioeconomic status (poverty income ratio) and child’s intake of added sugars were also taken into account, providing additional insights into the associations. Three sequential models were also fitted to test the association between obesity and caries experience. A two-sided *p* < 0.05 was considered statistically significant. 

## 3. Results

Among the 29,902 individuals who participated in the 2011–2016 NHANES survey, 4559 fell within the age range of 6–12 years. In total, 348 children who were underweight were removed from this group. Furthermore, 82 participants lacked data on both body measurements and oral health examination, and an additional 806 were missing covariate information. Consequently, the final dataset for analysis comprised 3323 participants (see the flowchart in Figure 1).

The DMFT score was 1.69 (SD = 2.61), and the prevalence of untreated dental caries was 19.2%. The mean age was 8.90 (SD = 2.00) years, and boys accounted for 50.6% of the sample. A one-way ANOVA revealed that there was no statistically significant difference in mean age between boys and girls (*p* > 0.05).

Bivariable analysis of untreated dental caries is shown in Table 1. Gender was not associated with this outcome, while the remaining socioeconomic variables were associated with the prevalence of untreated dental caries. Mexican American children (23.7%), as well as those living in families with low socioeconomic status (24.3%), had a higher prevalence of untreated dental caries. 

Table 1 also shows the outcome of the bivariable analysis of the DMFT score. Boys (mean = 1.81, SD = 2.72) had higher DMFT scores on average. Differences in race/ethnicity and the poverty income ratio were also significant. Mexican American children (mean = 2.14, SD = 2.92), as well as those living in families with low socioeconomic status (mean = 2.12, SD = 2.81) and those with the highest intake of added sugars (mean = 1.93, SD = 2.82), had higher DMFT scores (Table 1). 

The prevalence of obesity based on the CDC standards was 38.2%. Obesity was more common among Mexican American children (48.6%) and those with low family socioeconomic status (41.2%) (Table 2).

The association of obesity with untreated dental caries and caries experience is shown in Table 3. In crude models, obese children had 1.163 (95%CI: 0.975,1.387) times more untreated caries than children with normal weight. This association was weakened after adjusting for child demographic factors (1.128, 95%CI: 0.943, 1.350). Further adjustments for the poverty income ratio and the child’s intake of added sugars resulted in a marginal decrease in the association (1.125, 95%CI: 0.939, 1.347). However, statistical significance was not reached. Notably, the association between obesity and caries experience was not significant in either unadjusted or adjusted models.

## 4. Discussion

In this study, school-aged children’s oral health status is reported based on dental caries experience and untreated dental caries. These are formative years when children are impressionable and need to develop learned lifestyle habits. Children’s oral health knowledge is based on surface level indicators, such as experiencing pain, rather than understanding caries and how to maintain good oral health on their own. It has been assumed that overconsumption and a child’s body measurements provide a direct assessment of dietary habits. Varied dietary intakes among school children. This variation are likely to reflect available diets; the values and circumstances of parents, schools, and peers; and their motivation. Childhood obesity and caries are extremely complex diseases with multifactorial causes and associated demographic factors. Race, socioeconomic status, diet, regular oral hygiene, fluoride intake, dental visits, disability, gender, and environment are all significant.

Being conducted in the U.S., this study introduces the variables of highly processed foods, increased sugar intake, and increased obesity in adolescents, thus diverging from international research on this topic. Children of all BMIs and household incomes can experience dental caries. However, in this study, low socioeconomic status and being Mexican American were each directly correlated with obesity, untreated dental caries, and higher DMFT scores. DMFT scores were also higher in boys and those with a higher intake of added sugars. A previous study found that low socioeconomic status bears a more significant relationship with obesity than race. They found that for every 1% increase in low income, obesity increases by 1.17% [28]. However, another study found that Mexican Americans had the highest rates of obesity regardless of socioeconomic status [29]. Both results align with the findings that low socioeconomic status and being Mexican American are both correlated with obesity. The research finding that DMFT scores were higher in boys seems to contrast with the literature, as females have often been found to have higher rates of caries [30]. 

There was no relationship between dental caries and obesity in US school-aged children, emphasizing the intricate and multifaceted nature of this relationship. While some studies have suggested a possible link between the two conditions, others have not found a significant association. Notably, research indicates that a correlation between obesity and caries is evident in children with permanent dentition but not in those with mixed dentition [31]. Low socioeconomic status was identified as a shared risk factor for caries and obesity in another study [14]. Internationally, diverse findings add to the complexity of this issue. For instance, a Chinese study revealed severe caries in underweight children, suggesting an impact on development and growth [32]. Another study from China indicates a direct association between caries and higher BMI in high-income countries, whereas this correlation is not observed in low- and middle-income countries [7]. In Saudi Arabia, an inverse relationship between caries and high BMI was identified [33]. Meanwhile, a study in Mexico found no correlation between dental caries and overweight or obese children but noted a correlation with poor hygiene [34]. In England, research shows a linear relationship between obesity and childhood caries in white populations, with an inverse relationship in non-white populations, potentially attributed to higher sugar consumption among white individuals [35]. Finally, research in New Zealand reported no association between childhood obesity and dental caries, although the sample size was limited to 200 children [36]. These international research results provide further evidence that a multitude of factors drive both childhood caries and obesity.

Several factors contribute to the lack of a clear correlation between dental caries and obesity in certain studies. Firstly, dietary patterns play a role in both conditions, but the specific dietary factors influencing each may vary, making it challenging to establish a consistent relationship. Additionally, variations in oral hygiene practices and behaviors related to diet and physical activity among individuals may not be fully comprehended or adequately captured in some studies. Moreover, the diversity among populations introduces another layer of complexity, with different cultural, socioeconomic, and regional factors influencing the prevalence of dental caries and obesity. Study design and methodology, including variability in sample size and the approach to controlling confounding factors, can contribute to conflicting findings. The evolving nature of the relationship over time, influenced by changes in dietary patterns, oral health practices, and lifestyle factors, further complicates the issue. Different age groups may also exhibit varying associations between dental caries and obesity, with some studies potentially missing developmental stage variations.

It is important to note that research in this field is ongoing, and new studies may provide additional insights. Additionally, health disparities and individual variations can contribute to differing outcomes in different populations. Therefore, it is challenging to make broad generalizations about the relationship between dental caries and obesity in all school-aged children. Researchers continue to explore these connections to better understand the multifaceted nature of oral health and obesity in pediatric populations.

### 4.1. Implications

The present findings have implications for policy and research. Understanding the intricacies that affect both caries and obesity, and understanding how they are related, will help drive policy to improve the health of the population. Differing results from international research on the topic show that location and culture undoubtedly play a role. In the US, further research on this topic may elucidate what public health interventions are needed and identify populations most vulnerable to obesity and caries. 

### 4.2. Limitations of the Study

Limitations of this study need to be discussed as well. First, the sampling methods categorized children into weight categories based on the BMI scale, a flawed method for determining if children are at a healthy weight. BMI does not differentiate lean mass (muscle and bone) from fat and has been known to misclassify individuals as either obese or non-obese. BMI is also less accurate compared to percent body fat analysis in classifying men and African Americans, often overestimating rates of obesity in these demographic categories [37]. Misclassifying participants based on a flawed BMI metric may have altered the study results. Ideally, future studies would measure body fat percentage in place of BMI as a more reliable indicator of obesity. An additional limitation of this study was the method of collecting sugar consumption data. Sugar consumption was determined based on recall of the patients’ previous 24 h food consumption. The accuracy of these data relied on parents’ honesty, as well as the ability to recall consumption accurately. Thus, data collected from parents can be misreported or inaccurate. 

A key advantage of this study is that it furthered research on caries risk in American school children, which broadens the knowledge of risk factors and demographics as they relate to caries experience. These results can be clinically applied by making clinicians more aware of populations that are more directly affected by caries and obesity. Dietary counseling and oral hygiene instruction can be tailored to at-risk populations.

Risk factors for poor oral health and childhood obesity should be further researched to improve both, as both are significant issues impacting children. In addition, prior research has shown that malnutrition can significantly affect oral health. Further research on dietary habits and how proper nutrition in children is correlated with caries experience and untreated caries is needed. Finally, studies relating poor hygiene and poor nutrition to an increased risk of dental caries may show that how children are parented has a significant impact on oral health. Family patterns, lifestyle, and cultural beliefs greatly impact how children learn about hygiene and nutrition and take care of their bodies. These factors serve as possible determinants of the correlation between obesity and dental caries, deserving prominent attention in subsequent studies. 

## 5. Conclusions

No statistically significant correlation was found between dental caries and obesity. Whether or not a correlation exists, it is recommended that future preventive programs focus on raising awareness about healthy dietary habits. This approach aims to address common risk factors and control both conditions.

## Figures and Tables

**Table 1:** Prevalence of untreated caries and mean DMFT score according to sociodemographics in NHANES cycles 2011–2016.

	*n* (%)	Untreated Caries		DMFT	
		*n* (%)	*p*-Value *	Mean (SD)	*p*-Value
Total	3323 (100)	6 37 (19.2)	NA	1.69 (2.61)	NA
Sex					
Boys	1682 (50.6)	341 (20.3)	0.07	1.81 (2.72)	0.02 ^a^
Girls	1641 (49.4)	296 (18.0)		1.57 (2.49)	
Race and ethnicity					
Mexican American	691 (20.8)	164 (23.7)	<0.001	2.14 (2.92)	<0.001 ^b^
Non-Hispanic Asian	249 (7.5)	48 (19.3)		1.78 (2.65)	
Non-Hispanic Black	865 (26.0)	180 (20.8)		1.74 (2.59)	
Non-Hispanic White	912 (27.4)	142 (15.6)		1.38 (2.39)	
Other Race ^1^	214 (6.4)	28 (13.1)		1.35 (2.40)	
Other Hispanic	392 (11.8)	75 (19.1)		1.62 (2.53)	
Ratio of family income to poverty					
≤1	1136 (34.2)	276 (24.3)	<0.001	2.12 (2.81)	<0.001 ^a^
>1	2187 (65.8)	361 (16.5)		1.46 (2.47)	
Child intake of added sugars					
Q1	829 (24.9)	155 (18.7)	0.91	1.49 (2.40)	0.02 ^b^
Q2	829 (24.9)	161 (19.4)		1.73 (2.74)	
Q3	831 (25.0)	166 (20.0)		1.60 (2.44)	
Q4	834 (25.1)	155 (18.6)		1.93 (2.82)	

^1^ Including multi-racial. * Chi-square test. ^a^ Mann–Whitney U test. ^b^ Kruskal–Wallis test.

**Table 2:** Prevalence of obesity according to sociodemographics in NHANES cycles 2011–2016.

	*n* (%)	Obesity	
		*n* (%)	*p*-Value *
Total	3323 (100)	1270 (38.2)	NA
Sex			
Boys	1682 (50.6)	632 (37.6)	0.87
Girls	1641 (49.4)	638 (38.9)	
Race and ethnicity			
Mexican American	691 (20.8)	336 (48.6)	<0.001
Non-Hispanic Asian	249 (7.5)	61 (24.5)	
Non-Hispanic Black	865 (26.0)	328 (37.9)	
Non-Hispanic White	912 (27.4)	294 (32.2)	
Other Race	214 (6.4)	78 (36.4)	
Other Hispanic	392 (11.8)	173 (44.1)	
Ratio of family income to poverty			
≤1	1136 (34.2)	468 (41.2)	<0.001
>1	2187 (65.8)	802 (36.7)	
Child intake of added sugars			
Q1	829 (24.9)	337 (40.7)	0.59
Q2	829 (24.9)	306 (36.9)	
Q3	831 (25.0)	319 (38.4)	
Q4	834 (25.1)	308 (36.9)	

* Chi-square test.

**Table 3:** Models of the association of BMI groups with the DMFT index and prevalence of untreated caries.

	*n* (%)	Model 1 ^1^	Model 2 ^2^	Model 3 ^3^
		OR [95% CI]	OR [95% CI]	OR [95% CI]
Untreated Caries				
Normal	375 (58.9)	Reference	Reference	Reference
Obese	262 (41.1)	1.163 [0.975, 1.387]	1.128 [0.943, 1.350]	1.125 [0.939, 1.347]
Caries Experience		RR [95% CI]	RR [95% CI]	RR [95% CI]
Normal	N/A	Reference	Reference	Reference
Obese	N/A	0.919 [0.765–1.103]	0.899 [0.749–1.080]	0.899 [0.749–1.078]

^1^ Model 1 was unadjusted. ^2^ Model 2 was adjusted for demographic factors (sex, age, race/ethnicity). ^3^ Model 3 was additionally adjusted for family socioeconomic status (poverty income ratio) and child intake of added sugars (quintiles).

## Data Availability

The data for this study are freely available at https://wwwn.cdc.gov/nchs/nhanes/Default.aspx (accessed on 23 May 2023).

## Abbreviations

NHANES	National Health and Nutrition Examination Survey
CDC	Centers for Disease Control and Prevention
DMFT	decayed, missing and filled teeth
US	United States
MEC	mobile examination center
BMI	body mass index
FPED	Food Patterns Equivalent Database
USDA	US Department of Agriculture
tsp. eq.	teaspoon equivalent
OR	odds ratios
RR	rate ratios
